# Characteristic Metabolic Changes of the Crust from Dry-Aged Beef Using 2D NMR Spectroscopy

**DOI:** 10.3390/molecules25133087

**Published:** 2020-07-07

**Authors:** Hyun Cheol Kim, Ki Ho Baek, Yoon-Joo Ko, Hyun Jung Lee, Dong-Gyun Yim, Cheorun Jo

**Affiliations:** 1 Department of Agricultural Biotechnology, Center for Food and Bioconvergence, and Research Institute of Agriculture and Life Science, Seoul National University, Seoul 08826, Korea; eoenhc@naver.com (H.C.K.); kihoback@naver.com (K.H.B.); leehj0113@snu.ac.kr (H.J.L.); 2 National Center for Inter-University Research Facilities, Seoul National University, Seoul 08826, Korea; yjko@snu.ac.kr; 3 Institute of Green Bio Science and Technology, Seoul National University, Pyeongchang 25354, Korea

**Keywords:** 2D qNMR, wet aging, dry aging, crust, metabolomics, ^1^H-^13^C HSQC

## Abstract

Two-dimensional quantitative nuclear magnetic resonance (2D qNMR)-based metabolomics was performed to understand characteristic metabolic profiles in different aging regimes (crust from dry-aged beef, inner edible flesh of dry-aged beef, and wet-aged beef striploin) over 4 weeks. Samples were extracted using 0.6 M perchlorate to acquire polar metabolites. Partial least squares-discriminant analysis showed a good cumulative explained variation (*R*^2^ = 0.967) and predictive ability (*Q*^2^ = 0.935). Metabolites of crust and aged beef (dry- and wet-aged beef) were separated in the first week and showed a completely different aspect in the second week via NMR-based multivariable analyses. Moreover, NMR-based multivariable analyses could be used to distinguish the method, degree, and doneness of beef aging. Among them, the crust showed more unique metabolic changes that accelerated proteolysis (total free amino acids and biogenic amines) and inosine 5′-monophosphate depletion than dry-aged beef and generated specific microbial catabolites (3-indoxyl sulfate) and γ-aminobutyric acid (GABA), while asparagine, glutamine, tryptophan, and glucose in the crust were maintained or decreased. Compared to the crust, dry-aged beef showed similar patterns of biogenic amines, as well as bioactive compounds and GABA, without a decrease in free amino acids and glucose. Based on these results, the crust allows the inner dry-aged beef to be aged similarly to wet-aged beef without microbial effects. Thus, 2D qNMR-based metabolomic techniques could provide complementary information about biochemical factors for beef aging.

## 1. Introduction

Meat aging is a series of biological and physiochemical transitions from muscle to meat after slaughter that causes proteolysis and enhances meat tenderness and palatability [1,2]. These changes could be used to improve meat quality under controlled environments, such as temperature, relative humidity, aging period, and the aging method by packaging conditions [3]. The major aging methods are divided into dry and wet aging. Meat for wet aging is vacuum-packed, while meat for dry aging is exposed to air without packaging [4]. Dry-aged meat is known to generate concentrated flavors (beefy and roasted) after aging, while wet-aged meat is known to induce sour and bloody/metallic flavors [4,5,6].

Dry-aged meat is commonly produced in aerobic conditions, and its surface is dried rapidly [7]. The dried meat surface is called the crust. Significant water loss occurs in both the crust and internal meat during dry aging [3]. In general, the crust is trimmed-off and considered inedible because of not only hardness and dryness but a high concentration of microorganisms, such as mold and yeast [4,8,9]. Trimming and water losses of dry-aged meat are directly related to economic loss, even if it can create value-added meat after aging [10]. Lee et al. [3]. suggested that the microorganisms on the surface of dry-aged beef might be the primary reason for the differences between wet- and dry-aged beef, and the composition of the microorganisms may have a pivotal role in the production of characteristic flavors and textures [8]. Therefore, it could be hypothesized that these environmental differences may affect the metabolic changes of the crust and edible portion of dry-aged beef, followed by characteristic sensory properties.

For a quality assessment of beef products, chemometric analyses are required to measure various metabolites, such as free amino acids, peptides, nucleotides, sugars, lipids, and aromatic compounds [11]. Among various chemometric analyses, nuclear magnetic resonance (NMR) spectroscopy and/or chromatography-based mass spectroscopy have been widely used for elucidating metabolic profiles from various biological samples together with multivariable analyses [12]. Between them, NMR can provide intact chemical information without derivatization [13]. Several NMR studies combined with multivariable analyses have been reported on the metabolic profiles of during beef aging [14,15]. However, to our knowledge, there are no studies reported about metabolic changes in the crust of beef during the aging period.

Therefore, a two-dimensional quantitative NMR (2D qNMR) analysis was performed to understand characteristic metabolic changes in the crust, dry-aged, and wet-aged beef striploin after aging for 4 weeks.

## 2. Results and Discussion

The differences among dry- and wet-aged beef and the crust over 4 weeks of aging were investigated using 2D NMR-based metabolomics. For the comparison of polar metabolites in meat samples, perchloric acid extraction was performed [13]. Based on the 2D NMR spectra, metabolites were qualified and quantified for multivariable analyses.

### 2.1. Multivariable Analyses

**Figure 1:**
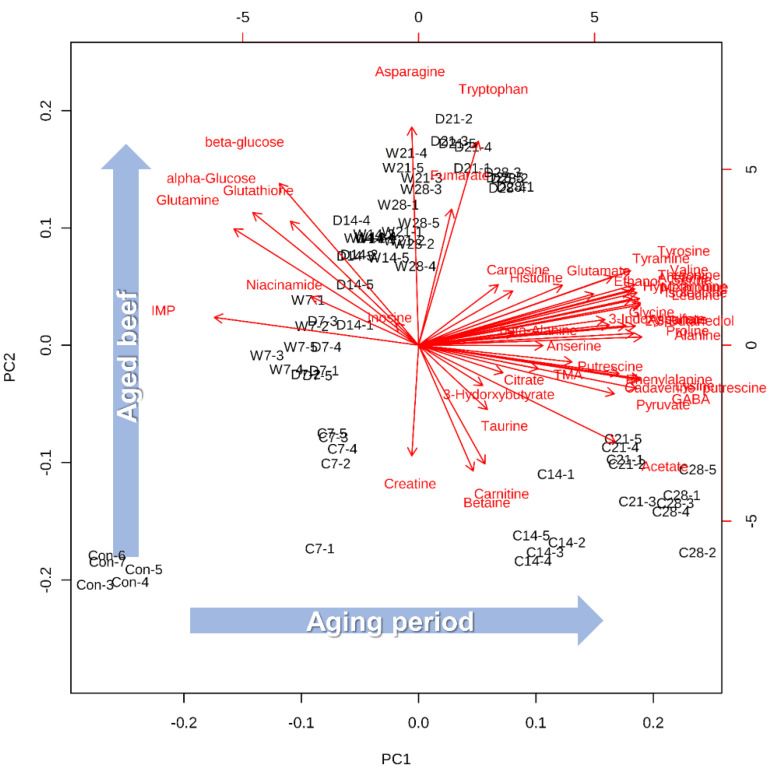
Principal components analysis (PCA) biplot (score plots and loadings of variable) from quantified metabolites of beef aged by different methods and crust. GABA, γ-aminobutyric acid; TMA, trimethylamine; IMP, inosine 5′-monophosphate.

Overall metabolic differences were determined using principal components analysis (PCA) based on the peak intensities from ^1^H-^13^C hetero single nuclear coherence (HSQC) NMR spectra (Figure 1). PCA is usually used to discriminate differences of samples composed of multivariable datasets via variance maximization and dimension reduction [16]. As seen in Figure 1, each meat sample was separated clearly by the aging period, along with PC1 (54.5%). Among all groups, the crust was more strongly correlated with PC1 than the others (wet- and dry-aged beef). However, the crust was not correlated with PC2 (12.5%) during the aging period, while dry- and wet-aged beef correlated with PC2 depending on the aging period. Dry- and wet-aged beef showed a similar appearance in the PCA. However, dry-aged beef was better-differentiated by PC1 than wet-aged beef. Wet-aged beef was hard to be distinguished between 21 and 28 days. Along with the PC2, dry- and wet-aged beef exhibited a linear correlation until 21 days, and then the correlation decreased by 28 days of the aging period.

The loading plots of variables, which visualize variables, can be used for understanding metabolic patterns easily [17]. Most of the metabolites were associated with PC1 and were composed of most free amino acids, biogenic amines, and bioactive compounds, except for some free amino acids (asparagine, tryptophan, and glutamine), inosine 5′-monophosphate (IMP), α- and β-glucose, and glutathione (Figure 1). The proportional increase in free amino acids and their derivatives (biogenic amines and bioactive compounds), depending on the aging period, was a well-known result of the aged meat [18]. Biogenic amines such as cadaverine, putrescine, tyramine, and trimethylamine (TMA) commonly increased depending on the aging period, and these compounds are usually considered indicators of freshness in meat samples [19,20]. Moreover, not only IMP and α- and β-glucose are recognized as freshness indicators, but also these metabolites play an important role in meat flavor development during the cooking process [3,21]. In addition to PCA, partial least squares-discriminant analysis (PLS-DA) was performed to separate between groups of observations (*R*^2^ = 0.967, *Q*^2^ = 0.935). PLS-DA, which is called the supervised method, is similar to PCA, but it can discriminate differences based on each group [16]. In this study, PLS-DA showed a good cumulative explained variation (*R*^2^) and predictive ability (*Q*^2^), which meant that the different aging methods and their aging period were clearly distinguished based on the polar metabolic characteristics.

**Figure 2:**
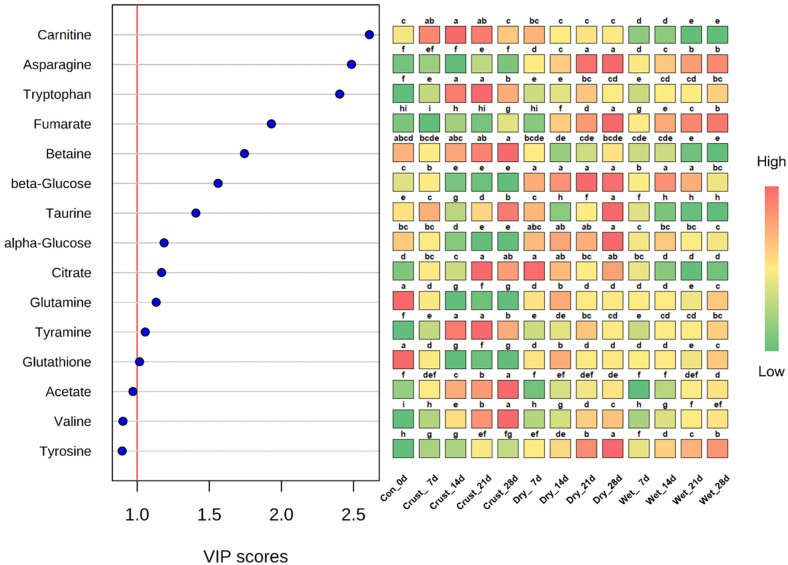
Variable importance in projection (VIP) scores of partial least squares-discriminant analysis (PLS-DA) from the extracts of crust, dry-aged, and wet-aged beef. The colored boxes on the right indicate the relative concentrations of the corresponding metabolites (red, high; yellow, intermediate; green, low). ^a–i^ Letters different in the same row indicate a significant difference (*p* < 0.05).

PLS-DA score plots showed similar appearances compared to PCA (Figure S1). Since PCA is considered an indicator of the practical reliability of PLS-DA, a similar appearance can explain that the data are clearly distinguished [16]. In PLS-DA, variables can be measured by scores, which are called variable importance in projection (VIP). The overall average of VIP scores is 1, and the metabolites above 1 contribute to the formation of the PLS-DA model [22]. Based on the VIP scores, twelve metabolites (>1 score) are presented in Figure 2. Three metabolites—carnitine, asparagine, and tryptophan—were the most characteristic variables to separate the groups, and these compounds were highly correlated in PC2 of PCA. Asparagine and tryptophan had a positive correlation with PC2, but carnitine had a negative correlation (Figure 1). Along with PCA, the positive correlation was related to the aged beef groups (dry- and wet-aged beef), and the negative correlation was related to the crust. The appearances of the metabolites could be the best way to distinguish between the aging method (dry- and wet-aged beef) and between the edible portion of aged beef and crust. Two other metabolites—fumarate and betaine—were also related to aged beef groups and crust, respectively. Other high VIP score metabolites were related to aged beef (β-glucose, α-glucose, glutamine, and glutathione) and crust (taurine, citrate, and tyramine).

**Figure 3:**
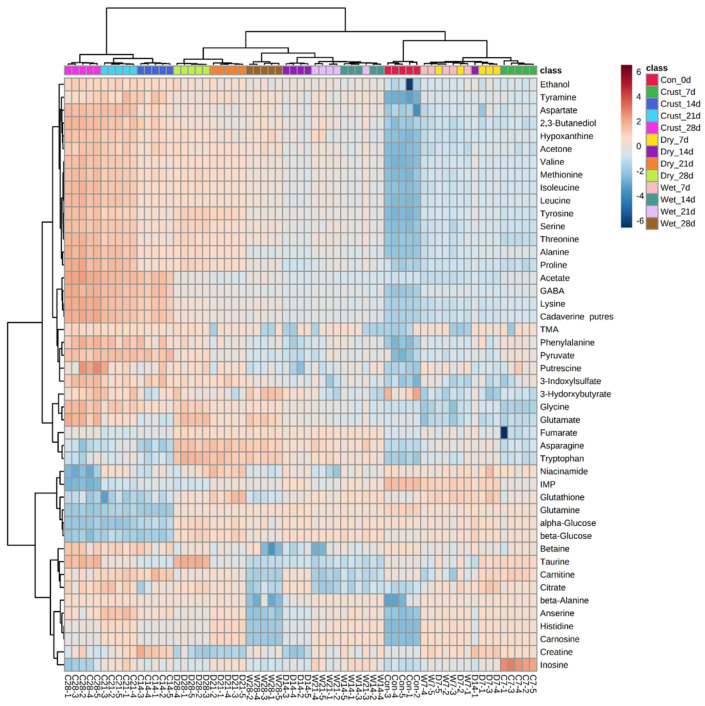
Heatmap analysis based on the quantified metabolites from crust, dry-aged, and wet-aged beef using 2D NMR (heteronuclear single quantum coherence, HSQC) on 850 MHz cryo-NMR spectrometer. GABA, γ-aminobutyric acid; TMA, trimethylamine; IMP, inosine 5′-monophosphate.

A hierarchical clustering heatmap analysis was performed for the classification between different observations (Figure 3). This method arranges the groups based on the similarity of the datasets and can be easily recognized using a dendrogram and heatmap [23]. As seen in the dendrogram, the greatest differences were shown between the crust and the other groups after the second week. The differences were clearly expressed in the PCA (Figure 1). These noticeable differences in the crust could be attributed to water evaporation and microbial effects [3,8,24]. Among the aged beef, most groups were well clustered by group except for some regions where the first week of dry- and wet-aged beef and the second week of dry-aged beef were mixed and the second and third weeks of wet-aged beef were mixed. Based on the results, the metabolic characteristics were clearly differentiated in the crust and dry-aged beef in the second week. In a previous study, similar results were reported that the unique flavor of dry-aged beef needs to age at least 2 weeks [3].

### 2.2. Metabolic Characteristics

#### 2.2.1. Proteolysis

Regardless of the treatment, total free amino acids were significantly increased, depending on the aging period (Table 1). The increase in amino acids and biogenic amines is generally proportional to the aging period [18]. Free amino acids are directly related to meat taste and/or contribute to flavor formation during cooking [3,25,26]. Total free amino acids were highest in the crust, then dry-aged, and finally wet-aged beef. In the first week of aging, total free amino acids did not differ significantly between the crust and dry-aged beef, while wet-aged beef was lower than the others (*p* < 0.05). The total free amino acids of the crust began to increase significantly after the second week of aging. The increase in free amino acids in the crust may be due to steady water evaporation during dry aging and accelerated proteolysis by microorganisms [3].

In dry aging, tryptophan in the crust increased in the third week and decreased in the fourth week. Unlike dry-aged beef, glutamine in the crust rapidly decreased during the first 2 weeks (*p* < 0.05). The increase in total free amino acid and the decrease in tryptophan and glutamine are closely related to proteolysis and catabolism of microorganisms, respectively [27,28]. Asparagine and glutamine could be degraded during storage via deamination when it is contaminated by microorganisms [27]. Moreover, tryptophan in the crust increased slightly during aging, but the rate of increase was lower than dry- or wet-aged beef, while 3-indoxyl sulfate steadily increased significantly from the second and third weeks in the crust and dry-aged beef, respectively (*p* < 0.05; Figure 3). However, in wet-aged beef, 3-indoxyl sulfate did not differ significantly after the first week. Tryptophan can be degraded into 3-indoxyl sulfate, which was reported as uremic toxin, provisionally related to glomerular sclerosis, tubulointerstitial fibrosis, oxidative stress in endothelial cells, and vascular endothelial cell dysfunction by microorganisms [28].

Biogenic amines showed similar trends to free amino acids (Figure 1 and Figure 3). Not only free amino acids increased, but also biogenic amines such as cadaverine, putrescine, tyramine, and TMA commonly increased depending on the aging period, and these compounds are also usually considered as indicators of freshness in meat samples [18,19]. Biogenic amines increased in the crust and dry-aged beef in the second and third weeks, respectively. As described above, PC1 scores of cadaverine, putrescine, tyramine, and TMA were 0.20, 0.14, 0.17, and 0.11, while PC2 scores were −0.06, −0.03, 0.13, and −0.04, respectively. Except for tyramine, biogenic amines could be used for the identification of crust.

#### 2.2.2. Bioactive Compounds

**Figure 4:**
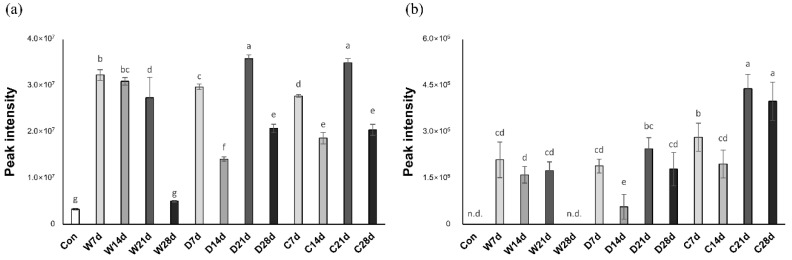
Peak intensities of (**a**) histidine and (**b**) β-alanine from different aged beef striploin (*longissimus lumborum*) using ^1^H-^13^C HSQC on 850 MHz cryo-NMR spectrometer. ^a–g^ Different letters in the same row indicate a significant difference (*p* < 0.05). n.d., not detected.

Anserine in the crust increased up to the third week and decreased during the fourth week, while that in wet-aged beef increased rapidly in the second week and decreased during the aging period (*p* < 0.05; Table 2). Anserine in dry-aged beef tends to increase and decrease significantly every two weeks of aging. Carnosine showed the same pattern as anserine in the crust and dry-aged beef, but in the case of wet-aged beef, it tended to increase significantly during the first week of aging and then decrease as the aging period increased. The changes of anserine and carnosine during the aging period and the tendency of histidine and β-alanine were consistent (Figure 4). Histidine can be used as a carbon source with glucose during microbial energy metabolism [29]. Compared to β-alanine, histidine concentration is closely related to histidyl dipeptides concentration [30]. The changes in fluctuated histidyl dipeptides could be affected by changing the major strain during the aging period in which initial bacteria increased during the first two weeks and did not change greatly in a later period, while lactic acid bacteria, yeast, and mold increased steadily [31]. Anserine and carnosine are histidyl dipeptides containing β-alanine, which has similar bioactivities, such as a buffering effect and antioxidant activity, to histidyl dipeptides, chelate metal ions, or scavenge free radicals [32]. Moreover, histidyl peptides are associated with anti-aging, antiglycation, neurotransmitter functions, and the alleviation of diseases, such as Alzheimer’s disease, cataracts, diabetes, and ischemia [33]. Carnosine also contributes to the umami taste in meat [34]. Regardless of treatment groups, anserine and carnosine decreased in the fourth week (*p* < 0.05). However, Fu et al. [18] reported that the bioactivities of wet-aged beef increased proportionally by aging period.

Carnitine is associated with fatty acid-related energy-generating processes, enhancing the utilization of fatty acids, and facilitating the removal of accumulated fatty acids from mitochondria. Thus, it could be advantageous for human health and help with weight loss [35]. Carnitine in the crust steadily increased up to the third week and then decreased (*p* < 0.05). Carnitine in dry- and wet-aged beef increased up to the first week and remained stable, while that in wet-aged beef decreased during the first week of aging and then remained stable (*p* < 0.05). Carnitine can be synthesized from lysine and methionine [36]. The decrease in carnitine during aging might contribute to the elevation of TMA by microorganisms, and it could be increased at the terminal stage of dry aging [3,37]. Creatine increased in the first week in all groups. Creatine increased until the second week and decreased in the third week in the crust (*p* < 0.05). In dry-aged beef, creatine decreased in the second week. On the other hand, wet-aged beef maintained creatine during the aging period. Creatine is related to muscle energy metabolism and provides energy during exercise [38]. Phosphocreatine rapidly converts to creatine postmortem [39].

#### 2.2.3. Nucleotides

The major nucleotides (IMP, inosine, and hypoxanthine) of beef samples are presented in Table 3. Most of ATP in muscles at postmortem is dephosphorylated into AMP and is then converted to IMP, which contributes to an umami taste and imparts flavor to the meat. IMP is converted to inosine and then to hypoxanthine, which contributes to the bitter taste [34]. Regardless of the aging method, the IMP contents gradually decreased, while the hypoxanthine contents increased as aging length increased (*p* < 0.05). Similarly, IMP contents in the crust rapidly decreased in the first week of aging and were significantly lower than those in the dry- and wet-aged beef during the aging period.

Dry- and wet-aged beef had no differences at the first 2-weeks in the IMP contents. However, the IMP contents of dry-aged beef were higher in the third week and decreased significantly in the fourth week compared to those of wet-aged beef (*p* < 0.05). A similar trend has been observed in previous studies [3,40]. This decrease in dry-aged beef could be associated with enzymes related to the degradation of IMP [3]. IMP was mainly produced at the initial postmortem and degraded during the aging period [34]. Yamaguchi [41] reported that a certain ratio of IMP and glutamic acids showed a strong synergistic effect on the umami taste. However, this lower IMP of dry-aged beef compared to wet-aged beef in the fourth week did not cause a difference in the umami taste of dry-aged beef in this study. In the crust, inosine increased in the first week and decreased in the fourth week of aging, and hypoxanthine was proportional during the aging period regardless of aging methods. As described above, the PC1 scores of IMP, inosine, and hypoxanthine were −0.18, −0.02, and 0.19, while the PC2 scores were 0.05, 0.04, and 0.09, respectively (Figure 1). Nucleotides are used as freshness indicators, called the *k*-index (%) and biogenic amines index, that develop during proteolysis [20]. Based on the nucleotides, the crust rapidly decreased in freshness compared to dry- and wet-aged beef.

### 2.3. Unique Metabolic Characteristics of the Crust during Aging

The crust showed a higher concentration of citrate, pyruvate, ethanol, acetone, 2,3-butanediol acetate, and 3-hydroxybutyrate, while both α-glucose and β-glucose were lower than in dry- and wet-aged beef (*p* < 0.05; Figure 3). The increased compounds during aging are catabolites of microorganisms through glucose metabolism with glucose as substrates [42]. Along with the decrease in glucose, fumarate also decreased, while citrate increased. Therefore, it is shown that microorganisms grown on the outer surface of beef (crust) during aging may play a vital role, affect metabolic changes inside the meat, and significantly influence final meat qualities [8,24,43]. Moreover, it is found that γ-aminobutyric acid (GABA) increased on the crust from the second week of aging and increased on the dry-aged beef at the fourth week of aging compared to wet-aged beef (*p* < 0.05). GABA is usually produced during the fermentation of yeast and fungi [44] and is known to have functional effects, such as lowering blood pressure [45]. Previous results demonstrated that the degradation of myofibrillar proteins is not only achieved by endogenous proteolytic enzymes but also by exogenous microbial proteolytic action during the dry aging process [3,8,44].

Despite the beneficial effect, harmful compounds such as biogenic amines and 3-indoxyl sulfate can also be generated in the crust during dry aging (Figure 3). The amounts of glucose, glutamine, asparagine, tryptophan, and fumarate were higher in dry- and wet-aged beef than those in the crust during the second week of aging. This difference could be explained by the fact that both dry- and wet-aged beef were less influenced by microorganisms as yeast and mold cannot penetrate the crust itself [46]. On the contrary, Peromingo et al. [47] suggested fungal secondary metabolites on the surface of dry-cured meat diffused into the meat. Moreover, a previous study reported that mold and yeast growth on the surface of dry-aged beef and their composition (ratio) directly affected meat sensory properties [8].

## 3. Materials and Methods

### 3.1. Sample Preparation and the Aging Process

A total of 60 striploins (*longissimus lumborum*) from both sides of 30 different beef carcasses (21-month-old Holstein steers, quality grade 3) were obtained at 48 h postmortem from a local slaughterhouse and transferred to the meat processing plant (Seoul, Korea). The striploin from the same sides of different carcasses were randomly arranged in each dry- and wet-aged group (Control was randomly selected additionally). Before the aging process, the wet-aged group was vacuum-packaged (HFV-600 L, Hankook Fujee Co., Ltd., Hwaseong, Korea) with a low-density polyethylene/nylon bag (oxygen permeability of 22.5 mL/m^2^/24 h atm at 60% relative humidity (RH)/25 °C and water vapor permeability of 4.7 g/m^2^/24 h at 100% RH/25 °C). Twenty beef samples were randomly selected for both groups and aged for 7, 14, 21, and 28 days under different conditions. Considering the terminal weight loss of dry-aged beef, 1 kg of striploins was aged on a wire rack with the fat surface facing down at 4 °C, 75% RH, and 2.5 m/s airflow velocity. Wet-aged samples (500 g of striploins) was aged at 4 °C after vacuum-packaging. At the sampling stage, the crust of the dry-aged beef was trimmed off approximately 1 cm from the external surface of each dry-aged meat. The crust, dry-, and wet-aged beef samples were vacuum-packaged (HFV-600 L, Hankook Fujee Co., Ltd., Hwaseong, Korea) and stored at −70 °C until the analyses.

### 3.2. Sample Extraction

Frozen beef samples were thawed at 4 °C for 24 h before analysis. Thawed beef samples (5 g) were homogenized at 1720× *g* for 30 s (T25 basic, Ika Co., KG, Staufen, Germany) with 0.6 M perchloric acid. The homogenate was centrifuged (Continent 512R, Hanil Co., Ltd., Incheon, Korea) at 3086× *g* for 15 min. The supernatant was transferred to a new test tube and neutralized with potassium hydroxide. Neutralized extracts were centrifuged again under the same conditions. After centrifugation, each supernatant was filtered using filter paper (Whatman No. 1, Whatman PLC., Middlesex, UK) and lyophilized (Freezer dryer 18, Labco Corp., Kansas City, MO, USA). The lyophilized extracts were stored at −70 °C until NMR analysis.

### 3.3. NMR Experiments

One-dimensional (1D) ^1^H NMR and ^1^H-^13^C HSQC were recorded in deuterium oxide (heavy water; D_2_O) at 298 K on a Bruker 850 MHz cryo-NMR spectrometer (Bruker Biospin GmbH, Rheinstetten, Germany). One-dimensional ^1^H NMR was performed applying a modified standard zg30 (recycle delay of 1 s) pulse sequence provided by default in Topspin 3.6.2 (Bruker Biospin GmbH, Rheinstetten, Germany), with the lock on the deuterium resonance. The 1D ^1^H NMR experiment was performed with 64k data points and a sweep width of 17,006.803 Hz with 128 scans. The ^1^H-^13^C HSQC experiment was performed as follows: 2k data points in the t_2_ domain and 512 increments in the t1 with 8 scans; spectral widths of 11 ppm for the f2 dimension and 180 ppm for the f1 dimension; and coupling constant values of 145 Hz were employed to set delay durations for short-range correlations, respectively. ^1^H-^13^C HSQC spectra were also used for qualification. The chemical shifts (δ) were referenced to the 3-(trimethylsilyl)propionic-2,2,3,3-*d*_4_ acid (TSP) resonance. Baseline correction was performed manually.

### 3.4. Multivariable Analysis

The dataset [65 (samples) × 46 (metabolites) matrix] of the acquired integral data of each metabolite from ^1^H-^13^C HSQC was collected using AMIX (Analysis of MIXtures software v3.9, Bruker Biospin GmbH, Rheinstetten, Germany). The data were analyzed using one-way ANOVA with Tukey’s post hoc test where *p* < 0.05 was considered to be significant. PCA biplot, VIP scores of PLS-DA, and heatmap analysis were performed using MetaboAnalyst 4.0 (www.metaboanalyst.ca) according to Xia and Wishart [48]. Prior to the analysis, samples were log-transformed and auto-scaled. Heatmap analysis was evaluated using Euclidean distance and ward cluster algorithm.

### 3.5. Quantification of Metabolites

Peaks of the metabolites were identified based on the standard compounds and biological magnetic resonance bank (BMRB; bmrb.wisc.edu). Quantification (totally 46 metabolites) was calculated based on the ^1^H-^13^C HSQC spectra using AMIX (Bruker Biospin GmbH, Rheinstetten, Germany; Figure S2). Prior to quantification, 1D ^1^H NMR spectra were acquired and processed according to Kim et al. [13] to quantify 24 metabolites using an internal standard of 1 mM TSP. Calibration curves were made with the peak intensities from ^1^H-^13^C HSQC (Figure S3).

### 3.6. Statistical Analysis

Statistical analysis was performed using the procedure of the general linear model for the comparison of quantified metabolites. Significant differences among mean values were determined by Student–Neuman–Keul’s multiple range test using SAS software (SAS 9.4, SAS Institute Inc., Cary, NC, USA) with a confidence level of *p* < 0.05. All the experimental procedures were conducted in quintuplicate.

## 4. Conclusions

Based on this study, the metabolic appearance of crust, dry-, and wet-aged beef were separated from the first week and showed a completely different aspect from the second week, as shown by NMR-based multivariable analyses. Moreover, NMR-based multivariable analyses could be used for distinguishing the aging degree. Among them, the crust exhibited unique metabolic changes that accelerated proteolysis (total free amino acids and biogenic amines) and IMP depletion than in dry-aged beef, and generated specific microbial catabolites (3-indoxyl sulfate) and GABA, while asparagine, glutamine, tryptophan, and glucose were maintained or decreased. Compared to the crust, dry-aged beef showed similar patterns of biogenic amines and bioactive compounds and GABA without a decrease in free of amino acids and glucose. Based on these results, the crust allows the inner dry-aged beef to be aged similarly to wet-aged beef. Moreover, the metabolic changes of the crust could affect the metabolites of dry-aged beef. However, understanding how external conditions affect dry-aged beef is necessary to produce high value-added beef products.

## Figures and Tables

**Table 1:** Free amino acids contents (mg/100 g) from different aged beef striploin (*longissimus lumborum*) using ^1^H-^13^C HSQC on 850 MHz cryo-NMR spectrometer.

Contents	Aging Method	Aging Period					SEM ^1^
		0	7	14	21	28	
Alanine	Crust	15.95 ^e^	27.26 ^d,x^	37.91 ^c,x^	47.19 ^b,x^	52.95 ^a^^,^^x^	0.852
	Dry	15.95 ^d^	26.25 ^c,x^	26.52 ^c,y^	36.35 ^b,y^	39.93 ^a,y^	0.264
	Wet	15.95 ^d^	23.30 ^c,y^	27.35 ^b,y^	27.92 ^b,z^	29.42 ^a,z^	0.312
	SEM ^2^		0.359	0.339	0.517	0.980	
Asparagine	Crust	3.79 ^b^	4.69 ^ab,y^	3.30 ^b,y^	5.49 ^a,z^	3.95 ^b,z^	0.363
	Dry	3.79 ^d^	7.07 ^c,x^	8.63 ^b,x^	13.23 ^a,x^	13.59 ^a,x^	0.388
	Wet	3.79 ^d^	6.94 ^c,x^	9.30 ^b,x^	11.02 ^a,y^	11.96 ^a,y^	0.481
	SEM ^2^		0.448	0.445	0.518	0.428	
Aspartic acid	Crust	1.39 ^d^	4.11 ^c^	13.37 ^b,x^	25.22 ^a,x^	25.15 ^a,x^	0.699
	Dry	1.39 ^d^	3.77 ^c^	5.71 ^b,y^	7.87 ^a,y^	9.14 ^a,y^	0.585
	Wet	1.39 ^d^	3.54 ^c^	7.80 ^b,y^	8.66 ^ab,y^	9.82 ^a,y^	0.462
	SEM ^2^		0.654	0.589	0.630	0.625	
Glutamic acid	Crust	6.74 ^e^	27.25 ^d,x^	67.31 ^a,x^	62.59 ^b,x^	56.80 ^c,x^	0.844
	Dry	6.74 ^e^	12.88 ^d,y^	18.53 ^c,y^	23.25 ^b,y^	30.44 ^a,z^	0.489
	Wet	6.74 ^e^	11.95 ^d,y^	17.84 ^c,y^	24.92 ^b,y^	35.83 ^a,y^	0.855
	SEM ^2^		0.369	0.934	1.013	0.865	
Glutamine	Crust	49.09 ^a^	34.14 ^b^	6.10 ^d,z^	8.12 ^c,z^	6.07 ^d,z^	0.553
	Dry	49.09 ^a^	36.12 ^c^	42.98 ^b,x^	34.71 ^c,x^	35.46 ^c,y^	0.688
	Wet	49.09 ^a^	35.05 ^c^	35.21 ^c,y^	32.16 ^d,y^	39.22 ^b,x^	0.781
	SEM ^2^		0.828	0.327	0.381	0.567	
Glycine	Crust	25.09 ^d^	21.16 ^e,y^	35.55 ^c,x^	40.28 ^b,x^	44.72 ^a,x^	0.761
	Dry	25.09 ^d^	25.83 ^d,x^	31.83 ^c,y^	34.15 ^b,y^	39.66 ^a,y^	0.450
	Wet	25.09 ^c^	21.65 ^d,y^	28.46 ^b,z^	30.40 ^b,z^	36.09 ^a,z^	0.702
	SEM ^2^		0.832	0.702	0.664	0.700	
Isoleucine	Crust	3.77 ^e^	10.08 ^d,x^	21.79 ^c,x^	30.62 ^b,x^	37.25 ^a,x^	0.616
	Dry	3.77 ^e^	10.69 ^d,x^	13.58 ^c,y^	20.80 ^b,y^	23.60 ^a,y^	0.188
	Wet	3.77 ^e^	9.07 ^d,y^	13.93 ^c,y^	16.78 ^b,z^	18.32 ^a,z^	0.290
	SEM ^2^		0.228	0.396	0.253	0.736	
Leucine	Crust	7.21 ^e^	17.79 ^d,x^	31.11 ^c,x^	43.72 ^b,x^	50.38 ^a,x^	0.821
	Dry	7.21 ^e^	17.76 ^d,x^	20.58 ^c,z^	30.07 ^b,y^	32.42 ^a,y^	0.301
	Wet	7.21 ^d^	15.83 ^c,y^	22.63 ^b,y^	25.70 ^a,z^	26.83 ^a,z^	0.519
	SEM ^2^		0.257	0.569	0.580	0.989	
Methionine	Crust	4.43 ^e^	12.34 ^d,y^	18.68 ^c,x^	23.50 ^b,x^	27.08 ^a,x^	0.408
	Dry	4.43 ^c^	13.77 ^b,x^	14.56 ^b,z^	21.61 ^a,y^	21.67 ^a,y^	0.344
	Wet	4.43 ^d^	12.41 ^c,y^	16.51 ^b,y^	18.48 ^a,z^	16.75 ^b,z^	0.283
	SEM ^2^		0.285	0.364	0.430	0.416	
Proline	Crust	8.56 ^d^	9.89 ^d,y^	16.97 ^c,x^	23.66 ^b,x^	27.18 ^a,x^	0.627
	Dry	8.56 ^d^	10.72 ^c,x^	11.23 ^c,z^	17.98 ^b,y^	19.30 ^a,y^	0.375
	Wet	8.56 ^d^	10.58 ^c,x^	12.89 ^b,y^	14.11 ^ab,z^	14.65 ^a,z^	0.435
	SEM ^2^		0.188	0.353	0.491	0.683	
Serine	Crust	6.61 ^e^	9.92 ^d,y^	18.18 ^c,x^	24.04 ^b,x^	27.00 ^a,x^	0.481
	Dry	6.61 ^e^	11.73 ^d,x^	14.58 ^c,y^	19.93 ^b,y^	22.07 ^a,y^	0.155
	Wet	6.61 ^e^	10.19 ^d,y^	14.55 ^c,y^	16.32 ^b,z^	17.92 ^a,z^	0.322
	SEM ^2^		0.358	0.321	0.309	0.511	
Taurine	Crust	45.68 ^d^	56.38 ^b,x^	39.30 ^e,x^	48.02 ^c,x^	67.42 ^a,y^	0.620
	Dry	45.68 ^c^	55.03 ^b,x^	36.44 ^e,y^	43.50 ^d,y^	71.26 ^a,x^	0.692
	Wet	45.68 ^a^	41.56 ^b,y^	34.27 ^c,y^	34.26 ^c,z^	33.98 ^c,z^	0.883
	SEM ^2^		0.698	0.911	0.689	0.930	
Tryptophan	Crust	3.91 ^c^	5.26 ^b,y^	5.29 ^b,y^	6.78 ^a,z^	5.76 ^b,z^	0.343
	Dry	3.91 ^d^	6.86 ^c,x^	7.71 ^c,x^	10.92 ^b,x^	12.39 ^a,x^	0.303
	Wet	3.91 ^e^	6.45 ^d,x^	8.04 ^c,x^	9.32 ^b,y^	10.54 ^a,y^	0.232
	SEM ^2^		0.347	0.220	0.274	0.414	
Valine	Crust	3.18 ^e^	9.56 ^d,x^	17.94 ^c,x^	26.14 ^b,x^	30.92 ^a,x^	0.582
	Dry	3.17 ^e^	9.88 ^d,x^	12.51 ^c,y^	19.48 ^b,y^	20.76 ^a,y^	0.256
	Wet	3.37 ^e^	8.99 ^d,y^	13.34 ^c,y^	16.06 ^b,z^	16.90 ^a,z^	0.263
	SEM^2^		0.109	0.388	0.348	0.702	
Total free amino acids	Crust	200.89 ^e^	280.30 ^d,x^	386.52 ^c,x^	478.71 ^b,x^	533.85 ^a,x^	6.282
	Dry	200.89 ^e^	279.48 ^d,x^	301.81 ^c,y^	383.80 ^b,y^	446.23 ^a,y^	2.958
	Wet	200.89 ^e^	246.77 ^d,y^	300.74 ^c,y^	330.32 ^b,z^	360.88 ^a,z^	3.759
	SEM ^2^		3.884	4.278	4.084	7.102	

^1, 2^ Standard error of the means ^1^ (*n* = 25), ^2^ (*n* = 15). ^a–e^ Different letters in the same row indicate a significant difference (*p* < 0.05). ^x–z^ Different letters in the same column indicate a significant difference (*p* < 0.05).

**Table 2:** Bioactive compounds (mg/100 g) from different aged beef striploin (*longissimus lumborum*) using ^1^H-^13^C HSQC on 850 MHz cryo-NMR spectrometer.

Contents	Aging Method	Aging Period (Day)					SEM ^1^
		0	7	14	21	28	
Anserine	Crust	19.93 ^c^	90.26 ^b,y^	95.08 ^b,x^	147.98 ^a,x^	96.89 ^b,x^	0.852
	Dry	19.93 ^d^	100.21 ^a,x^	53.12 ^c,y^	98.30 ^a,y^	72.07 ^b,y^	0.264
	Wet	19.93 ^c^	92.04 ^a,y^	91.40 ^a,x^	78.30 ^b,z^	22.72 ^c,z^	0.312
	SEM ^2^		2.438	3.932	3.193	5.267	
Betaine	Crust	15.36 ^ab^	14.78 ^b^	16.34 ^ab,x^	17.15 ^ab,x^	17.67 ^a,x^	0.363
	Dry	15.36 ^a^	14.76 ^ab^	13.58 ^b,y^	14.19 ^ab,y^	14.98 ^ab,xy^	0.388
	Wet	15.36	14.15	14.18 ^y^	13.08 ^y^	12.93 ^y^	0.481
	SEM ^2^		0.302	0.637	0.546	0.873	
Carnitine	Crust	39.61 ^c^	44.95 ^ab,x^	46.01 ^a,x^	45.35 ^ab,x^	42.70 ^b,x^	0.699
	Dry	39.61 ^b^	43.41 ^a,x^	41.44 ^ab,y^	41.76 ^ab,y^	41.24 ^ab,x^	0.585
	Wet	39.61 ^a^	34.97 ^b,y^	34.87 ^b,z^	32.52 ^b,z^	32.47 ^b,y^	0.462
	SEM ^2^		0.866	0.642	0.451	0.650	
Carnosine	Crust	33.97 ^d^	418.83 ^b,z^	256.24 ^c,y^	498.79 ^a,y^	260.74 ^c,y^	0.844
	Dry	33.97 ^e^	471.15 ^b,y^	205.82 ^d,z^	546.28 ^a,x^	297.89 ^c,x^	0.489
	Wet	33.97 ^e^	499.34 ^a,x^	476.09 ^b,x^	455.32 ^c,z^	53.02 ^d,z^	0.855
	SEM ^2^		5.908	6.005	5.221	5.655	
Creatine/Phosphocreatine	Crust	182.13 ^c^	215.28 ^b,y^	239.46 ^a,x^	205.85 ^bc,x^	192.74 ^b,c^	0.553
	Dry	182.13 ^b^	227.47 ^a,x^	167.30 ^b,y^	150.83 ^b,y^	157.55 ^b,y^	0.688
	Wet	182.13 ^b^	211.49 ^a,y^	200.98 ^ab,xy^	202.34 ^ab,x^	197.01 ^ab,x^	0.781
	SEM ^2^		3.806	12.983	6.496	9.142	

^1, 2^ Standard error of the means ^1^ (*n* = 25), ^2^ (*n* = 15). ^a–e^ Different letters in the same row indicate a significant difference (*p* < 0.05). ^x–z^ Different letters in the same column indicate a significant difference (*p* < 0.05).

**Table 3:** Nucleotide contents (mg/100 g) from different aged beef striploin (*longissimus lumborum*) using ^1^H-^13^C HSQC on 850 MHz cryo-NMR spectrometer.

Contents	Aging Method	Aging Period (Day)					SEM ^1^
		0	7	14	21	28	
IMP	Crust	135.21 ^a^	57.42 ^b,y^	46.52 ^c,y^	36.54 ^d,z^	10.22 ^e,z^	1.104
	Dry	135.21 ^a^	99.78 ^b,x^	80.49 ^c,x^	66.71 ^d,x^	38.29 ^e,y^	1.291
	Wet	135.21 ^a^	102.60 ^b,x^	75.64 ^c,x^	56.68 ^d,y^	53.05 ^d,x^	1.330
	SEM ^2^		1.154	1.612	1.094	0.953	
Inosine	Crust	16.04 ^d^	27.48 ^a,x^	18.58 ^c,y^	20.48 ^b^	14.93 ^d,y^	0.476
	Dry	16.04 ^d^	18.79 ^b,y^	17.59 ^c,y^	20.17 ^a^	17.74 ^c,x^	0.287
	Wet	16.04 ^c^	18.50 ^b,y^	20.24 ^a,x^	20.03 ^a^	16.81 ^c,x^	0.476
	SEM ^2^		0.408	0.423	0.589	0.400	
Hypoxanthine	Crust	12.54 ^e^	25.45 ^d,x^	37.67 ^c,x^	43.53 ^b,x^	46.64 ^a,x^	0.540
	Dry	12.54 ^e^	22.72 ^d.y^	27.64 ^c,y^	33.51 ^b,y^	37.36 ^a,y^	0.880
	Wet	12.54 ^d^	21.15 ^c,z^	26.90 ^b,y^	32.18 ^a,y^	36.04 ^a,y^	1.415
	SEM ^2^		0.502	1.006	0.253	0.765	

^1, 2^ Standard error of the means ^1^ (*n* = 25), ^2^ (*n* = 15). ^a–e^ Different letters in the same row indicate a significant difference (*p* < 0.05). ^x–z^ Different letters in the same column indicate a significant difference (*p* < 0.05).

## Supplementary Materials

The following are available online, Figure S1: Partial least square-discriminant analysis (PLS-DA) cross validation from quantified metabolites of beef aged by different method and crust. Validation was evaluated (a) overall groups, (b) dry- and wet-aged beef, (c) crust and dry-aged beef, and (d) crust and wet-aged beef, Figure S2: Representative ^1^H-^13^C hetero nuclear single quantum coherence (HSQC) NMR spectrum from 28 day-aged crust of dry aging extracts acquired using a 850 MHz cryo-NMR spectrometer and metabolites list of pattern integration for 2D qNMR analysis, Figure S3: Standard curves of quantification from ^1^H-^13^C hetero nuclear single quantum coherence (HSQC) based on the 1D ^1^H quantitative NMR using a 850 MHz cryo-NMR spectrometer.
