# Supplementary material for: Characteristic Metabolic Changes of the Crust from Dry-Aged Beef Using 2D NMR Spectroscopy
Source: Molecules. 2020 Jul 7;25(13):3087. doi: 10.3390/molecules25133087 (PMC7411603; doi:10.3390/molecules25133087)
Supplement: Supplementary file 1 [file molecules-25-03087-s001.pdf]

Supplementary Material

Characteristic Metabolic Changes of the Crust from Dry-aged Beef Using 2D NMR spectroscopy

Hyun Cheol Kim [1], Ki Ho Baek [1], Yoon-Joo Ko [2], Hyun Jung Lee [1], Dong-Gyun Yim [1,*] and Cheorun Jo [1,3,*]

[1] Department of Agricultural Biotechnology, Center for Food and Bioconvergence, and Research Institute of Agriculture and Life Science, Seoul National University, Seoul 08826, Korea; eoenhc@naver.com (H.C.K.); kihoback@naver.com (K.H.B.); leehj0113@snu.ac.kr (H.J.L.)

[2] National Center for Inter-University Research Facilities, Seoul National University, Seoul 08826, Korea; yjko@snu.ac.kr

[3] Institute of Green Bio Science and Technology, Seoul National University, Pyeongchang 25354, Korea

* Correspondence: tousa0994@naver.com (D.-G.Y.); cheorun@snu.ac.kr (C.J.); Tel.: +82-2-880-4820 (D.-G.Y.); Tel.: +82-2-880-4804 (C.J.)

Figure S1. Partial least square-discriminant analysis (PLS-DA) cross validation from quantified metabolites of beef aged by different method and crust. Validation was evaluated (a) overall groups, (b) dry- and wet-aged beef, (c) crust and dry-aged beef, and (d) crust and wet-aged beef.

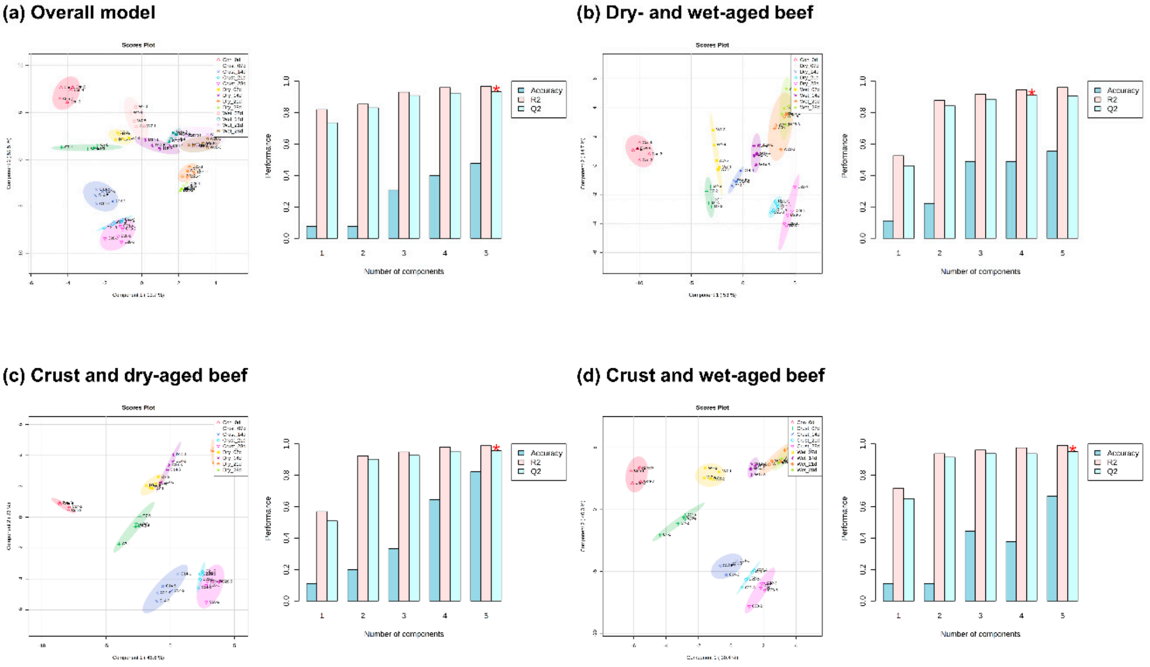

Figure S2. Representative ^{1}H-^{13}C hetero nuclear single quantum coherence (HSQC) NMR spectrum from 28 day-aged crust of dry aging extracts acquired using a 850 MHz cryo-NMR spectrometer and metabolites list of pattern integration for 2D qNMR analysis.

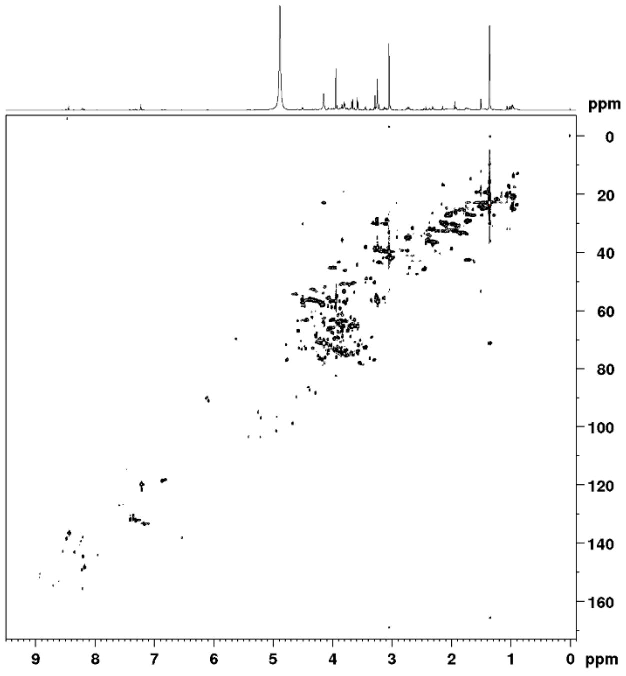

Compounds	Chemical shift (δ)			
	^{13}C		^{1}H	
	δ_{final}	$\delta_{initial}$	δ_{final}	$\delta_{initial}$
2,3-Butanediol	21.43	18.62	1.17	1.12
3-Hydroxybutyric acid	25.51	23.75	1.23	1.18
3-Indoxyl sulfate	115.61	113.32	7.50	7.41
Acetate	27.18	25.25	1.97	1.87
Acetone	33.34	31.58	2.24	2.17
Alanine	19.77	18.07	1.52	1.46
Anserine	29.52	27.59	3.12	2.99
Asparagine	38.14	36.44	2.99	2.85
Aspartic acid	40.00	38.46	2.78	2.69
Betaine	56.76	55.36	3.29	3.26
Cadaverine	26.10	24.23	1.50	1.42
Carnitine	46.96	44.25	2.50	2.38
Carnosine	31.58	29.65	3.12	2.99
Citrate	48.17	46.53	2.76	2.68
Creatine	40.59	38.48	3.06	3.00
Ethanol	20.25	18.65	1.21	1.16
Fumarate	139.85	137.22	6.56	6.48
Glutamic acid	58.21	55.22	3.83	3.70
Glutamine	34.63	32.52	2.51	2.40
Glutathione	34.79	33.21	2.62	2.51
Glycine	45.31	43.03	3.61	3.54
Histidine	31.73	29.09	3.27	3.17
Hypoxanthine	149.34	147.05	8.20	8.13
Inosine	91.88	90.30	6.10	6.06
Inosine 5'-monophosphate	88.12	86.72	4.41	4.35
Isoleucine	14.61	12.55	0.99	0.91
Leucine	25.53	22.72	1.00	0.93
Lysine	42.66	40.94	3.06	2.93
Methionine	17.94	15.62	2.16	2.07
Niacinamide	128.26	125.80	7.63	7.55
Phenylalanine	39.97	38.56	3.16	3.09
Proline	69.56	67.82	4.31	4.24
Putrescine	26.10	24.23	1.81	1.76
Pyruvic acid	30.37	28.97	2.38	2.35
Serine	63.89	61.97	4.03	3.94
Taurine	39.07	37.16	3.46	3.40
Threonine	64.06	61.98	3.65	3.57
Trimethylamine	47.96	46.56	2.91	2.88
Tryptophan	54.80	53.10	4.04	3.98
Tyramine	118.95	117.37	6.84	6.77
Tyrosine	119.45	117.71	6.89	6.83
Valine	21.91	19.75	1.09	1.02
α-Glucose	64.36	62.43	3.80	3.75
β-Alanine	37.08	35.49	2.60	2.54
β-Glucose	64.32	62.74	3.93	3.87
γ-Aminobutyric acid	37.08	35.50	2.32	2.25

Figure S3. Standard curves of quantification from ^{1}H-^{13}C hetero nuclear single quantum coherence (HSQC) based on the 1D ^{1}H quantitative NMR using a 850 MHz cryo-NMR spectrometer.

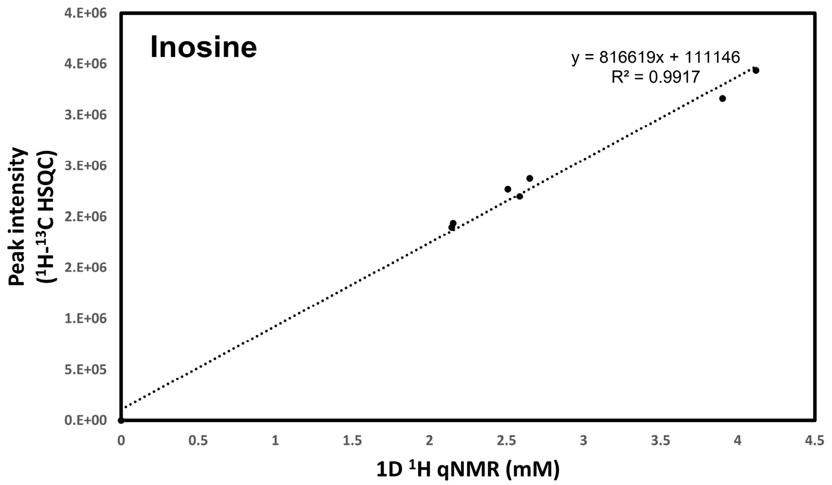

Compounds	R^2
Alanine	0.996
Anserine	0.998
Asparagine	0.999
Aspartic acid	0.997
Carnitine	0.981
Carnosine	1.000
Creatine	0.965
Glutamic acid	0.996
Glutamine	0.986
Glycine	0.968
Hypoxanthine	0.992
Inosine	0.992
Inosine 5'-monophosphate	0.990
Isoleucine	0.990
Leucine	0.994
Lysine	0.996
Methionine	0.996
Phenylalanine	0.996
Proline	0.968
Serine	0.998
Taurine	0.998
Tryptophan	0.997
Tyrosine	0.997
Valine	0.996
